# CRISPR/Cas9 technology in the modeling of and treatment of mucopolysaccharidosis

**DOI:** 10.1016/j.bbrep.2024.101771

**Published:** 2024-07-01

**Authors:** Mehran Reyhani-Ardabili, Soudeh Ghafouri-Fard

**Affiliations:** Department of Medical Genetics, Shahid Beheshti University of Medical Sciences, Tehran, Iran

**Keywords:** Mucopolysaccharidosis, MPS, CRISPR/Cas9

## Abstract

Mucopolysaccharidosis (MPS) syndromes are a group of heterogeneous genetic disorders in terms of genetic basis and clinical manifestations, ranging from mild to fatal forms. There are a number of applied or prospective treatment modalities for MPS, including bone marrow transplantation, enzyme replacement therapy, targeted gene therapy and substrate reduction therapy. Recently, CRISPR/Cas9 technology has emerged as a novel tool for several metabolic disorders, such as MPS. This review concentrates on the application of this technique in the treatment of MPS, particularly MPS I, and modeling of disease-causing mutations.

## Highlights

• CRISPR/Cas9 technology has emerged as a novel tool for MPS.
• This technique can revolutionize treatment of MPS, particularly MPS I.
• Moreover, this technique has been used for modeling of MPS-causing mutations.

## 1 Introduction

Mucopolysaccharidosis (MPS) syndromes are a group of heterogeneous genetic disorders comprising 7 types and 13 subgroups [1]. All of these conditions are caused by defects in the enzymes that degrade glycosaminoglycans (GAGs). These defects result in the widespread accumulation of GAGs within the lysosomes of different organs. Therefore, clinical manifestations can be detected in almost all body systems, such as eye, central nervous system, lung, heart, bone and the gastrointestinal system [2]. While some cases are presented with minor systemic and ocular defects and have a normal life span, other might have severe phenotypes resulting in the death in the first few months of life [1].

All types of MPS except for type II are inherited as autosomal recessive disorders [3]. Alternatively named as the Hunter's syndrome, MPS type II has an X-linked inheritance and is caused by the defects in the iduronate-2-sulfatase enzyme encoded by *IDS* gene on chromosome Xq28 [3]. Type I MPS is caused by mutations in *IDUA* gene, which is located on chromosome 4p16, and encodes the a-l-iduronidase enzyme. This type of MPS has three subtypes, namely Hurler, Hurler-Scheie, and Scheie syndromes, with the first subtype being the most severe subtype [4]. Accumulation of dermatan and heparan sulfates in MPS I occurs in various tissues and results in pervasive organ dysfunction [4].

Type III (Sanfilippo syndrome) has 4 subtypes, namely IIIA-IIID caused by mutations in the *SGSH*, *NAGLU*, *HGSNAT* and *GNS* genes, respectively [[5], [6], [7], [8]]. Type IV (Morquio syndrome) has two subtypes involving mutations in the *GALNS* and *GLB1* genes, respectively [9,10]. Finally, types VI, VII and IX are due to mutations in *ARSB* (chromosome 5q11), *GUSB* (chromosome 7q11), and *HYAL1* (chromosome 3p21) genes, respectively [1].

There are a number of treatment modalities, including bone marrow transplantation and enzyme replacement therapy to increase their life span of affected individuals and enhance their quality of life [1]. However, application of enzyme replacement therapy is limited by the challenges caused by crossing the blood–brain barrier [11]. Thus, this treatment is not applicable in the severe forms of MPS with the involvement of the central nervous system. Targeted gene therapy and substrate reduction therapy have also been suggested as prospective treatments for MPS [1]. Recently, CRISPR/Cas9 technology has emerged as a novel tool for several metabolic disorders [12]. This review focuses on the application of this technique in the treatment of MPS and modeling of disease-causing mutations.

## 2 CRISPR/Cas9 technique

Being firstly recognized as an RNA-mediated immune system, CRISPR/Cas system defends prokaryotes against bacteriophages and horizontal plasmid transmission [13]. Generally, the CRISPR/Cas systems are categorized into two main subtypes. While class 1 systems use multi-protein effector complexes, class 2 systems implement single-effector complexes making them more suitable for gene editing applications and screening [14]. The CRISPR/Cas9 system is an example of the second class, which has been broadly utilized for gene editing applications. Cas9 protein and guide RNA (gRNA) are the main apparatuses of the CRISPR/Cas9 system. In fact, Cas9 is a multi-domain DNA endonuclease capable of cleaving the target DNA and making double-strand breaks [15]. Mechanistically, gRNA is made by the combination of tracrRNA and crRNA. While the former serves as a binding scaffold for nuclease, the latter pairs with the target sequence and is responsible for its specificity [16]. Thus, gRNA can be programmed to provide the specificity of the CRISPR/Cas9 system. The action of this system is accomplished through three steps of recognition, cleavage and restoration of induced double strand breaks. The latter is performed by the host cellular system through non-homologous end-joining or homology-directed repair (HDR) pathway with the latter being more accurate [17]. Numerous *in vitro* and *in vivo* approaches have demonstrated applicability of CRISPR/Cas9 systems for modeling and treatment of different types of MPS.

## 3 CRISPR/Cas9 systems for modeling of different types of MPS

Several intracellular mechanisms contribute to the pathogenesis of different types of MPS. Although a number of related cascades have been identified, details about early cellular aberrations that lead to irreversible neuronal injury have not been elucidated. CRISPR/Cas9 technology can facilitate development of cellular models of MPS and identification of the cellular cascades leading to certain abnormalities. Badenetti et al. used this technology to develop two human neuronal cell lines with IDS loss of function. They designed sgRNA against *IDS* exon 4. The first clone had an 18 nucleotide deletion in the mentioned exon and the second one carried a 203 nucleotide deletion including twenty nucleotides of this exon. Neuronal cells carrying these mutations had no IDS enzymatic activity and exhibited high GAG storage which led to reduced differentiation, down-regulation of LAMP1 and RAB7 proteins, compromised lysosomal acidification and augmented lipid storage. Furthermore, one of the two clones exhibited low levels of the autophagic marker p62. However, none of them exhibited noticeable oxidative stress or mitochondrial morphological changes. Thus, impaired IDS activity was suggested to affect neuronal differentiation at cellular level [18].

## 4 CRISPR/Cas9 technology for treatment of MPS I

de Carvalho et al. used the CRISPR-Cas9 editing system for correction of the most common MPS I-related mutation. *In vitro* assessments revealed enhancement of IDUA activity and reduction of lysosomal mass in human fibroblasts homozygous for p.Trp402*. Moreover, the presence of wildtype sequence in these cells was confirmed by next generation sequencing, revealing the ability of CRISPR-Cas9 genome editing for correction of causative mutations in MPS I [19]. This treatment strategy was also tested *in vivo*. In a combined *in vitro* and *in vivo* experiment, Schuh et al. used cationic liposomes carrying the CRISPR/Cas9 plasmid and a donor vector for MPS I gene editing. These complexes could significantly increase IDUA activity and reduce lysosomal abnormalities in fibroblasts of MPS I patients. Besides, hydrodynamic injection of the liposomal complexes in newborn MPS I mice resulted in significant enhancement of serum IDUA level. These complexes were detected in the lungs and heart. Besides, cardiovascular parameters were improved in animals after treatment with the liposomal formulation [20].

In another CRISPR/Cas9-based experiment, *IDUA* was introduced in the hematopoietic stem cells under the govern of a robust ubiquitous promoter. This gene was hosted in the CCR5 safe-harbor locus. Transplantation of these cells into a mouse model of MPS I led to reduction, but not normalization of neuroinflammation, and elimination of GAG in the liver and spleen. Yet, this treatment did not lead to clearance of GAG from the brain. While some behavioral abnormalities were amended, working memory was lessened in the treated animals [21].

Ou et al. made a proprietary system that inserted a promoterless *IDUA* cDNA sequence into the locus encoding albumin in the hepatocytes. They used adeno-associated virus-8 (AAV8) vector for delivery of this system into neonatal and adult MPS I mice. They showed enhancement of IDUA enzyme activity in the brain, normalization of storage levels, and improvement of memory and learning ability as demonstrated by neurobehavioral tests. Besides, histological test showed the efficacy of this method in reduction of foam cells in the hepatic tissue and vacuolation in neurons. Their experiments caused no vector-associated toxicity, no increased tumorigenesis and no off-target effects. The latter was confirmed through the unbiased genome-wide sequencing [22].

Ibraheim et al. described AAV structures that express Nme2Cas9 and either two sgRNAs, or a single sgRNA. While the former was used for segmental deletion, the latter was designed to be a template for HDR. They also used anti-CRISPR proteins to permit self-inactivation of vectors via Nme2Cas9 cleavage. The designed strategy was able to treat MPS I in mice through HDR-based editing method. Authors concluded that single-vector AAVs can be used to yield diverse therapeutic genome editing results [23].

Most notably, an experiment in mouse models of MPS I showed that nasal administration of liposomal complexes transporting two plasmids that encode the CRISPR/Cas9 system and the *IDUA* gene resulted in a moderate enhancement of IDUA activity in the lung, heart, and brain. Moreover, this treatment could reduce GAG concentrations in the serum, urine, tissues, and brain cortex. Besides, authors documented improvement in behavioral tests in the treated animals [24]. Table 1 shows the results of different attempts for MPS modeling using CRISPR/Cas9.

**Table 1:** MPS modeling using CRISPR/Cas9.

Cell line (In vitro)	Animal model (In vivo)	MPS type	CRISPR-Cas9-Targeted Gene (s)	Gene product	Targeted site of gene	Mutation type	CRISPR-Cas9 delivery/vectors	Verification of the gene disruption	Other verification tests	Enzyme Activity	Morphology/features after editing with CRISPR	References
Mouse-derived induced pluripotent stem cells (iPSCs)	–	MPSI	Idua	Α-l-iduronidase	Exon 6	Idua−/− transgenic mice were created by incorporating a neomycin resistance gene (Neor) into exon VI of the Idua gene. To rectify this, diverse crRNA inserts and donor templates with different homology arm lengths (97 bp, 213 bp, and 543 bp) were formulated for corrective genetic interventions.	–	Pluripotent Stem Cell Marker Immunofluorescent Staining	Stem Cell Gene Expression Validation, IDUA Activity Assay, Teratoma Formation Assay	Following CRISPR gene editing, Idua enzyme activity was notably restored, showing a substantial increase in the protein levels. There was no significant difference in Idua activity between the wild-type miPSC and the gene-corrected miPSC.	Wild-type and Idua−/− mouse iPSCs, along with control MEF-derived iPSCs, exhibited similar pluripotency markers, successful *in vitro* differentiation into ectoderm, mesoderm, and endoderm lineages, and no discernible differences in differentiation capabilities, suggesting unaffected iPSC derivation despite the absence of functional Idua enzyme.	[25]
Lund Human Mesencephalic (LUHMES) cell line	–	MPSII	IDS	Iduronate-2-sulfatase	Exon 4	Out of twenty mutagenized cell clones, a 50 % indel efficiency was observed, with over 20 % containing a 3-amino acid deletion, and clone 13 was identified as an IDS mutant with an 18-nucleotide deletion in exon 4.	Mixture of sgRNAs/Cas9	Sanger sequencing	RT-PCR, IDS enzymatic assay and measurement of GAG content, Fluorescent dye staining, Western blot, Mitotracker staining and electron microscopy	Cell lines show heightened GAG storage and lack of enzymatic activity despite having distinct genotypes.	Mutants exhibited reduced differentiation, lower levels of LAMP1 and RAB7 proteins, lysosomal acidification impairment, and increased lipid storage. One clone shows a notable decrease in the autophagic marker p62, while neither mutant displays significant oxidative stress or mitochondrial morphological alterations.	[26]
–	NSG mouse embryos (mouse NIH 3T3 cells)	MPSII	murine IDS gene	Iduronate-2-sulfatase	5′ UTR and downstream of exon 5 (intron 5)	–	A combination of both gRNAs and Cas9-encoding mRNA was injected into fertilized NSG mouse embryos using microinjection.	y Sanger sequencing of PCR product	Iduronate-2-sulfatase enzyme assay, Glycosaminoglycan assay, Skeletal analysis.	IDS-deficient NSG mice demonstrated no obvious IDS activity in plasma and tissues, alongside increased GAG levels in the analyzed tissues and urine.	The NSG-MPS II model mirrors skeletal abnormalities like enlarged zygomatic arch diameter and reduced femur length, alongside neurocognitive impairments in spatial memory and learning.	[27]
–	Zebrafish	MPSIIIA	sgsh	N-sulfoglucosamine sulfohydrolase	Exon 5,6	a 357 bp deletion	–	PCR of genomic DNA, Sanger sequencing	Sgsh Enzymatic Activity Assay,	The sgshΔex5-6 zebrafish mutant displays a total lack of Sgsh enzymatic activity.	The sgshΔex5-6 zebrafish model replicates CNS-specific MPS IIIA features, including neuroinflammation dependent on interleukin-1β, improved by Caspase-1 inhibition, and behavioral rescue.	[28]
–	Rat	MPSIVA	Galns	galactosamine (N-acetyl)-6-sulfatase	Exon11	c.1156C > T resulting in the p.Arg386Cys change	–	PCR and Sanger sequencing analyses	–	–	Selective backcrossing between Galns heterozygous and wild-type rats eliminated off-target effects, characterizing the MPSIVA rat model with features such as undetectable GALNS activity, elevated hepatic KS content, reduced body weight, shortened naso-anal length, and decreased tibial length, indicative of Morquio A disease.	[29]
–	C57BL/6J mice	MPSIVB	Mouse Glb1 gene	β-galactosidase	Exon 8	Among the live offspring, two founders carried a 2 bp mutation causing the W274L amino acid substitution, and another founder bore a 20 bp deletion	Microinjection of Cas9 protein and guide RNA (gRNA).	Sanger sequencing	β-galactosidase (β-gal) enzyme assay, Histopathological analysis, Ganglioside isolation and quantification,	Glb1^W274L^ mice exhibited a notable decrease in β-gal enzyme activity without displaying any significant phenotype after a year. On the other hand, β-gal−/− mice lacked β-gal enzyme activity leading to the buildup of gangliosides and extensive cellular vacuolation across the CNS.	β-gal−/− mice had severe neuromotor and neurocognitive dysfunction.	[30]
–	C57BL/6J mice	MPSVIB	Arsb	Aryl sulfatase B	Exon 1	Generation of Y58H knock-in mice. (c. 252T > C human ARSB mutation knock-in)	CUY21EDIT II (BEX)	Genotyping of generated mice	Micro-CT analysis, Measurement of ARSB activity, Colloid iron staining, Immunofluorescence microscopy, Western blotting	The liver and kidney extracts from both heterozygous and homozygous mice showed reduced ARSB enzyme activity, indicating that the 256T > C mutation affects the enzyme function.	The created mouse model displayed characteristics of MPS VI patients, such as facial features, mucopolysaccharide buildup, and reduced body size.	[31]

## 5 CRISPR/Cas9 technology for treatment of other types of MPS

Leal et al. showed the suitability of a CRISPR/Cas9 method for treatment of MPS IV through using a Cas9 nickase to insert an expression cassette encompassing *GALNS* cDNA into the AAVS1 locus in human fibroblasts of MPS IVA patients. Notably, they used iron oxide nanoparticles as non-viral vectors [32]. They experiments showed long-term expression of the desired gene, reduction of lysosomal mass, and amelioration of mitochondrial-derived reactive oxygen species in the mentioned fibroblasts [32]. Subsequently, they used the similar method in mouse model of MPS IVA through inserting the human *GALNS* cDNA into the *ROSA26* locus. They demonstrated improved *GALNS* activity, mono-keratan sulfate decrease, and partial amelioration of the bone pathology. Thus, they provided *in vivo* proof of the capacity of a CRISPR/nCas9-based method for treatment of MPS IVA using non-viral vectors [33].

As one of the pioneer studies in this field, extracellular vesicles were used to deliver CRISPR genome editing in *Gusb* mice as a mouse model of human MPS VII. AAV2-DJ vectors were administered to the mice through tail vein or retroorbital intravenous infusion. The latter treatment was able to improve both corneal transparency and survival of the experimental mice [34].

**Fig. 1:**
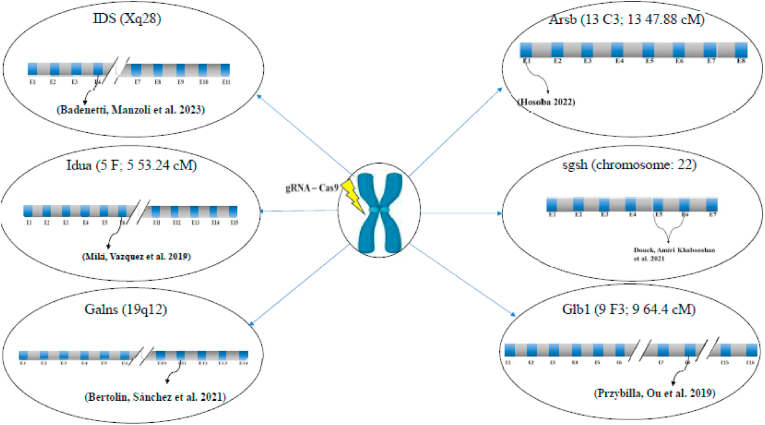
An overview of different MPS_related sites edited by CRISPR/Cas9 in different species.

Fig. 1 shows an overview of different MPS_related sites edited by CRISPR/Cas9 in different species.

Table 2 summarizes the results of studies that reported the application of CRISPR/Cas9 technology for editing of genes related to different types of MPS.

**Table 2:** Application of CRISPR/Cas9 technology for editing of genes related to different types of MPS.

Corrected/Engineered cells	Disease name	Gene	Insertion locus	Delivery system (with component)	Analysis of targeting efficiencies	Features of edited cells	Methodology Summary	References
Cord blood-derived (CB) and adult peripheral blood-derived HSPCs (PB)	Hurler syndrome	IDUA	The IDUA gene was integrated into the third exon of the CCR5 safe harbor loci.	The templates for homologous recombination featured IDUA expression units controlled by either SFFV or PGK promoters in the AAV vector, with YFP following a self-cleaving P2A peptide, along with an additional IDUA unit under PGK control lacking a selection marker.	YFP fluorescence, genotyping single cell-derived colonies from colony formation assays.	The engineered cells produce enzyme levels exceeding natural levels, exhibit sustained repopulation and differentiation capabilities, and show promise in ameliorating biochemical and phenotypic irregularities in an immunocompromised mouse model of MPS type I.	1 AAV donor plasmid construction 2 rAAV production 3 Electroporation and transduction of cells 4 Measurement of putative CCR5 gRNA off-target activity 5 Measurement of insertions at the CCR5 locus with ddPCR 6 HSPC selection and culturing 7 Colony forming unit assay and clonal genotyping 8 Macrophage differentiation and flow cytometry 9 Phagocytosis assay 10 Mice: Transplantation of CD34^+^ HSPCs into NSG mice 11 Assessment of human engraftment 12 IDUA activity assay 13 Analysis of GAGs using the DMB method and by LC/MS-MS 14 Histology 15 Immunocytochemistry 16 Computerized tomography	[35]
Brain and peripheral tissues in the mouse model of MPS I (nasal administration)	Hurler syndrome	The mouse Idua cDNA sequence controlled by an EF promoter and two homologous regions	The ROSA26 locus in mice, specifically within the region targeted by Cas9 for cleavage, which includes a copy of the hygromycin resistance gene	Liposomal complexes containing two plasmids—one encoding the CRISPR/Cas9 system and the other targeting the IDUA gene to the ROSA26 locus.	Assays to measure permeation and retention in porcine nasal mucosa, *in vivo* assay involving mice	In animal experiments, the therapy resulted in a slight rise in IDUA activity in the lung, heart, and brain regions, along with a decrease in GAG levels in serum, urine, tissues, and the brain cortex. Additionally, the treated mice displayed enhancements in behavioral assessments, indicating potential protection against cognitive impairment.	1 Vectors construction 2 Preparation of formulations 3 In vivo assays (Pilot (short-term) study, Extended (long-term) study) 4 IDUA activity assay 5 GAG levels assay 6 Cytokine levels assay 7 Behavioral tests	[24,36]
Human (skin) Fibroblasts Homozygous for p.Trp402* mutation	Hurler syndrome	IDUA	A 20-bp target sequence located near a NGG (PAM sequence),14 nucleotides away from the mutant base at p.Trp402*.	A 134 base single-stranded donor oligonucleotide homologous to the p.Trp402* region	Next generation sequencing, Enzyme assay, CytoPainter LysoDeep Red Indicator labeling	IDUA activity levels ranged from 4 % to 20 % (average 6 %) compared to normal fibroblasts. The treated MPS I cells exhibited a notable decrease in their lysosomal mass signal, with a reduction of around 25 % compared to the untreated MPS I group.	1 Vector construction 2 Cell culture and transfection 3 NGS 4 Enzyme assay 5 CytoPainter LysoDeep Red Indicator labeling	[19]
Fibroblasts from MPS1 patients	Hurler syndrome	IDUA	Designed construct with a 20-base pair target sequence near NGG site, 16 nucleotides from Trp402* mutation, containing mouse Idua cDNA under EF promoter regulation, two 1 Kb homologous regions for ROSA26 locus targeting, and a hygromycin resistance gene for clone selection experiments	PrecisionXTM® CRISPR/Cas9 SmartNuclease system, A specific oligonucleotide sequence corresponding to the p.Trp402* region was created, known as the donor oligonucleotide. The decision regarding the length of the homologous recombination (HR) arms and the selection of a single-stranded DNA (ssDNA) oligonucleotide as the HR template were as previous research. Using homology arms of 50–80 base pairs on either side of the mutation site yields the best outcomes.	–	Flow cytometry and confocal microscopy analyses revealed a decrease in the quantity of lysosomes to levels comparable to those found in normal cells, indicating a successful correction of the cellular phenotype.	–	[37]
Homozygous MPS-I IduaW392X mice	Hurler syndrome	Idua (mice)	Correction of the disease-causing G-to-A point mutation	Two Idua-targeting vectors containing Nme2Cas9, a sgRNA targeting Idua, and a 272-bp donor	PCR assays	–	–	[23]
CD34^+^ cells from healthy cord blood	Hurler syndrome	IDUA	Exon 3 of the CCR5 locus	–	HEX reference assay, Quantification of human/mouse DNA, Selection of IDUA-tNGFR + cells and flow cytometry	Prolonged engraftment and viability of bone marrow-derived cells within visceral organs and the central nervous system, leading to increased transgene expression and biochemical improvement in these tissues	–	[38]
Rat	Morquio A disease	GALNS	–	AAV expression cassettes were designed with either the rat Galns coding sequence or GFP cDNA under the control of the CAG promoter, with the GFP construct incorporating the WPRE element for enhanced protein production.	GFP fluorescence assay, Gene expression analysis, Vector genome copy number, Enzyme activities, Keratan sulfate quantification, Histology and electron microscopy, Bone structural analysis, Naso-anal length measurement, Grip strength	This resulted in a sustained elevation in GALNS activity and normalization of KS levels throughout the body, effectively averting reductions in body size and significant abnormalities in bones, teeth, joints, trachea, and heart.	–	[29]
NIH/3T3 mouse fibroblasts	Morquio A disease	GALNS	ROSA26	IONPs as non-viral vectors, lipofectamine LTX (LP-LTX)	–	In vitro validation demonstrated increased GALNS enzyme levels post-treatment, restoring lysosomal mass in MPS IVA fibroblasts to WT levels. Higher GALNS activity was observed in MTOL fibroblasts compared to human MPS IVA fibroblasts, with variations attributed to on-target Cas9 efficiency, showing the influence of sgRNA design and locus organization on CRISPR-nCas9-based gene editing efficacy.	1 Donor ROSA26 plasmid 2 On- and off-target evaluations 3 Homologous recombination assays 4 Lysosomal mass determination 5 GALNS activity	[33]
Human embryonic kidney (HEK293FT) cells	Morquio A disease	–	adeno-associated virus integration site (AAVS1) locus	AIO-mCherry plasmid, Lipofectamine 3000, sgRNA of dsDonor AAVS1:Empy or dsDonor AAVS1:GALNS	Sanger sequencing	The research displayed effective integration of the expression cassette into the AAVS1 locus, leading to a lasting elevation in GALNS activity, reaching levels as high as 40 % of normal. Furthermore, the study observed restoration of lysosomal mass, total GAGs, and mitigation of oxidative stress, pivotal discoveries concerning the pathophysiological mechanisms in MPS IVA.	1 Cell culture 2 Design and construction of plasmid vectors 3 CRISPR/nCas9 system validation 4 Genome editing on MPS IVA fibroblast	[39]

## 6 Discussion

Since MPS subtypes are monogenic diseases, gene therapy-based strategies are regarded as promising treatment methods as they are supposed to deliver long-term expressions of the desired transgene. The results of first clinical trial of genome editing via AAV-zinc-finger nucleases for MPS I/II showed promising safety profile with indications of targeted genome editing in liver. Yet, no long-term enzyme expression was documented in the blood [40]. Thus, this field awaits novel *in vivo* genome editing strategies or alternative delivery systems.

The implementation of CRISPR/Cas9 in MPS has raised the hope for the treatment of MPS, particularly for those who are not benefited from routine therapeutic options. Most of *ex vivo* and *in vivo* platforms have been examined in the contexts of MSP I, yet other types of MPS can also benefit from these techniques. *In vivo* application of these techniques awaits assessment of the safety of therapeutic genome editing systems [41]. These systems might induce modifications at unplanned genomic sites that could eventually lead to tumorigenicity. Short-lived Cas9 and mutant Cas9 with higher fidelity have shown to be effective in reduction of abrogation of this off-target problem [21,42,43]. Moreover, *in silico* tools have facilitated recognition of off-target sites. For instance, Carneiro et al. evaluated possible off-targets for a sgRNA targeting p.Trp402* as the most common variant detected in MPS I patients. They reported 272 potential off-target sequences as well as 84 polymorphic sites. Notably, most of polymorphic sites were predicted to reduce the probability of off-target cleavage. They also created a new PAM based on this analysis. Thus, off-targets should be screened in a population-specific context to increase safety and efficiency of CRISPR/Cas9-based methods [44].

Another challenging issue is the selection of desirable vectors. Although viral vectors have several benefits, their application is limited by some challenging issues, including the possibility of induction of immune response, oncogenesis activation, low package capacity, and vector dilution [45]. Non-viral vectors, such as iron oxide nanoparticles offer an alternative method with promising results in animal models [33]. Notably, these nanoparticles demonstrated high biocompatibility because they can be metabolized by the iron metabolism pathway [46]. Moreover, these nanoparticles may be proposed as a solution for the challenge encountered by the presence of neutralizing antibodies against Cas9 [33]. Table 3 summarizes some drawbacks when working with the CRISPR/Cas9 system and possible solutions.

**Table 3:** Drawbacks of the CRISPR/Cas9 system and possible solutions.

Drawbacks	Solutions
Off targeting	Application of bioinformatics tools for prediction and reduction of off-target modification, use of Cas9 nickase, adding anti-CRISPR proteins
Problems associated with viral vectors	Alternative delivery methods such as lipid nanoparticles or inorganic nanoparticles
Immunogenicity	Implementation of the CRISPR/Cas system early in the lifespan, injection of the editing system into the immune-privileged organs
Problems with editing efficiency	Enhancement of homology-directed repairmen, design of better guide RNAs
DNA-Damage Toxicity	Application of engineered Cas9 variants that do not cause double-stranded DNA breaks
Requirement for a PAM near the target site	RNA-targeting Cas9 variants like SpyCas9 with PAMmers, using CasRx that bypasses the need for a PAM sequence

Direct gene editing systems introduce only one functional copy of the desired gene into target cells, so they lack sufficient efficacy for treatment of diseases [47]. Overexpression the desired gene in hematopoietic stem cells is another strategy that might be used in future. Alternatively, application of a safe harbor locus to overexpress the desired gene has been suggested as a gene editing strategy in MPS. This approach led to promising results in the MPS I mouse models [21].

Taken together, CRISPR/Cas9 technique offers a promising method for the treatment of MPS. However, further assays using mice models of MPS are needed to test the real therapeutic effect of CRISPR/Cas9 approach in this type of metabolic disorders. Moreover, the minimum enzyme activity needed for amelioration of pathologic events in each tissue as well as the enzyme activity yield following different delivery methods should be determined. Besides, the presence of specific anti-Cas9 antibodies or cytotoxic T cells as well as the proinflammatory profile should be investigated in animal models treated with CRISPR/Cas9-based methods.

## Funding

No funding was received.

## CRediT authorship contribution statement

**Mehran Reyhani-Ardabili:** Data curation, Conceptualization. **Soudeh Ghafouri-Fard:** Writing – review & editing, Writing – original draft, Supervision, Data curation, Conceptualization.

## Declaration of competing interest

Authors declare no conflict of interests.
